# Patient-Reported Health-Related Quality of Life, Anxiety and Depression in Patients with Inclusion Body Myositis: A Register-Based Cross-Sectional Study in Germany

**DOI:** 10.3390/jcm12155051

**Published:** 2023-07-31

**Authors:** Katja C. Senn, Simone Thiele, Karsten Kummer, Maggie C. Walter, Klaus H. Nagels

**Affiliations:** 1 Chair of Healthcare Management and Health Services Research, University of Bayreuth, Parsifalstrasse 25, 95445 Bayreuth, Germany; katja.senn@uni-bayreuth.de; 2 Friedrich Baur Institute at the Department of Neurology, LMU University Hospital, LMU Munich, Ziemssenstrasse 1, 80336 Munich, Germany; simone.thiele@med.lmu.de (S.T.); maggie.walter@lrz.uni-muenchen.de (M.C.W.); 3 Department of Neurology, University Medical Center Goettingen, 37075 Goettingen, Germany; karsten.kummer@med.uni-goettingen.de

**Keywords:** anxiety, depression, quality of life, patient-reported outcome measures, inclusion body myositis

## Abstract

Inclusion body myositis (IBM) is a rare neuromuscular disease and the most prevalent idiopathic inflammatory myopathy (IIM) in patients aged older than 50 years. A systematic review has shown that no clear-cut conclusions can be drawn about the health-related quality of life (HRQoL) and mental health in IBM. We aimed to assess the HRQoL and mental health, to explore associated disease-related and socioeconomic factors as well as the utilization of psychological support in German IBM patients. This cross-sectional study included 82 patients registered in the German IBM patient registry. Patients had completed a survey battery including the EQ-5D-5L, the Individualized Neuromuscular Quality of Life (INQoL) and the Hospital Anxiety and Depression Scale German version (HADS-D). The physical HRQoL dimension was suggested to be most relevant. Most impaired life domains of HRQoL were mobility, independence, and activities. We identified significant differences in the total INQoL score for the degree of disability and care level as well as in depression for the degree of disability (*p* < 0.05), respectively. Most patients indicated no symptoms of anxiety (64.6%) and depression (62.2%). A more need-oriented psychological support in German IBM patients, reporting doubtful or definite anxiety or depression, could be suggested.

## 1. Introduction

Inclusion body myositis (IBM) is the most prevalent idiopathic inflammatory myopathy (IIM) in up to 139 per million patients older than age 50 [1,2,3]. This chronic disabling disease is progressing very slowly in contrast to other forms of myositis [4]. The typical age at onset is >45 years according to the definition of the latest diagnostic criteria of the European Neuromuscular Centre (ENMC). From a histopathology perspective, rimmed vacuoles combined with invading non-necrotic muscle fibers are a necessary pathological finding to confirm an IBM diagnosis [5]. Asymmetric, distal and proximal muscle weakness are further characteristics of the clinical phenotype [6]. Quadriceps femoris and finger flexors are predominantly affected and limit a patient’s physical functions such as climbing stairs, rising up from chairs or from the ground as well as holding and carrying objects [7,8,9]. In addition, dysphagia occurs frequently over the disease course and increases the risk of morbidity and mortality [10,11]. IBM patients mostly utilize walking aids (walking sticks or crutches) after a few years, and after approximately 10–16 years, they need wheelchairs [12,13]. Nevertheless, IBM per se does not shorten life expectancy, but thus far, causative therapies are not available [14]. However, high-dose intravenous immunoglobulins can slow down disease progression and are recommended in the German guidelines. These clinical practice guidelines recommend continuous physiotherapy, in order to maintain physical functioning and delay deteriorations, as well as symptomatic treatment of dysphagia [15]. Improvements on the actual care for IBM are needed, as data from the UK suggest insufficient referral rates and actual utilization of physiotherapy [16].

The patient-reported health-related quality of life (HRQoL) in IBM patients has been sparsely reported, mostly showing physical limitations [17]. For the other two dimensions within the construct of HRQoL, social and psychological HRQoL, clear conclusions cannot be drawn since the patient-reported outcome measures (PROMs) used and the respective outcome values varied [17]. Rare and chronic progressive diseases are associated with significant limitations in HRQoL as well as with increased psychiatric comorbidities such as anxiety or depression [18,19,20]. Accordingly, IBM patients are more likely exposed to a high risk of not receiving the required comprehensive health care services. Consequently, clinical outcomes are worse, which underpins the inevitable need for an in-depth and comprehensive understanding of HRQoL and the mental health state of patients diagnosed with neuromuscular diseases (NMD), and particularly in IBM [21,22,23].

For IBM patients treated in the German health-care setting, no comprehensive HRQoL or mental health data have been reported. Thus, it is not possible to compare or complement information regarding existing quantitative data from the UK, USA, Canada and Australia. Hence, the aim of this registry study was firstly, to describe the patient-reported outcomes (PROs) regarding mood disorders and HRQoL in patients from the German IBM registry (www.ibm-registry.org (accessed on 28 July 2023)) using generic and disease-specific PROMs; secondly, to explore disease-related and socioeconomic factors associated with anxiety, depression and HRQoL; lastly, to identify unmet needs or inadequate and ineffective use of support and health-care services. Moreover, the utilization of psychological support should be explored, considering self-reported anxiety and depression.

## 2. Materials and Methods

According to a sequential mixed-methods design, this quantitative study builds upon a preliminary systematic review about HRQoL and mental health [17], a qualitative interview study about HRQoL and a cost-of-illness (COI) study, in order to better understand the complex care situation of German IBM patients. Therefore, we conducted a cross-sectional survey study in 2021 and recruited IBM patients via the German IBM patient registry www.ibm-registry.org (accessed on 28 July 2023). In total, 111 patients were considered eligible if being diagnosed with IBM according to the ENMC diagnostic criteria [5,24], utilizing resources within the German health-care system and as being German speaking. We obtained ethical approval from the ethics committee of the Ludwig Maximilians University of Munich and considered the Helsinki Declaration. Written consent with the option of withdrawal was confirmed by all patients before completing the survey. The survey was primarily provided electronically, implemented via Qualtrics www.qualtrics.com (accessed on 28 July 2023), or on demand as a paper version. We gathered the ENMC diagnostic criteria [5] through the registry items and designed a questionnaire comprising a set of standardized generic and disease-specific PROMs together with items regarding demographics and socioeconomic characteristics.

### 2.1. Assessment of Physical Functioning, Comorbidities and Dysphagia

To better describe and explore the role of disease-related characteristics with regard to HRQoL and mood disorders, we firstly applied a German version of the sIBM Physical Functioning Assessment (sIFA) [25], the only available IBM-specific PROM regarding physical functioning. The sIFA comprises 11 items and is a numerical rating scale instrument (best to worst: 0–10). Secondly, we used the German Self-Administered Comorbidity Questionnaire (SCQ-D) [26,27] to assess the patient-reported comorbidities. The score of the SCQ-D (maximum 39) represents the extent of 13 predefined health problems (best to worst: 0–3 points each). Lastly, we measured dysphagia and applied the Sydney Swallow Questionnaire German version (SSQ-G) [28,29,30] as well as a German version of the Functional Oral Intake Scale (FOIS-G) [31]. The SSQ-G inventory assesses the self-reported oropharyngeal dysphagia globally (item 1), physically (items 2–16) and as the impact on the quality of life (item 17) with a visual analogue scale each (VAS; best to worst: 0–100), except the 6-point scale of item 12 [28]. Since the categorial FOIS-G is actually a clinician-reported outcome measure, we slightly adapted the wording of the seven possible levels more into everyday language to use it as a PROM.

### 2.2. Assessment of Generic and Disease-Specific HRQoL

The generic HRQoL was measured with the German version of the EuroQol five-dimension questionnaire (EQ-5D™ is a trademark of the Stichting EuroQol Research Foundation). The EQ-5D-5L assesses health states on a 5-point scale regarding patient-rated problems within five dimensions (mobility, self-care, usual activities, pain/discomfort and anxiety/depression) as well as with a VAS (EQ-5D VAS; best to worst: 0–100) [32]. We applied the single-index values for Germany to transform the health status into the respective estimated societal utility within the general population (range: −0.661–1.0) [33]. In addition, we applied the German version of the Individualized Neuromuscular Quality of Life (INQoL, version 2.0) [34] to measure the disease-specific HRQoL in NMD patients. This second revised INQoL version contains further items regarding the symptom impacts of ptosis, diplopia, and dysphagia. The INQoL is separated into the three sections symptom impact, the INQoL total score and treatment impact, whereby a higher calculated percentage of the scores indicates worse HRQoL.

### 2.3. Assessment of Anxiety and Depression

Though anxiety/depression is one part of the health state assessment of the EQ-5D, our aim was to measure these symptoms more precisely and apply a PROM, which could also serve as a feasible screening instrument for future clinical practice. Thus, we collected the PROs anxiety and depression by applying the German version of the Hospital Anxiety and Depression Scale (HADS-D) [35]. From two separate 7-item scales, two sum scales (anxiety: HADS-D/A; depression: HADS-D/D) could be calculated (best to worst: 0–21), whereby higher scales indicate a higher symptom burden. The data regarding resource utilization of psychological support were gathered from our preliminary COI study in this IBM patient cohort. We defined psychological support as every professional service such as psychosocial counselling, psychotherapy or behavioural therapy provided by social workers, counselling centres, psychotherapists or psychologists.

### 2.4. Analysis

We performed the statistical analysis using IBM© SPSS© Statistics version 28 (IBM, Armonk, NY, USA). Recommended procedures from the PROM developers were applied for the handling of missing data. Descriptive statistics were presented as median and interquartile range (IQR) as well as means and standard deviations to better compare the results with the existing data reported heterogeneously. The applied Shapiro–Wilk test indicated no normally distributed data. In order to analyze differences between the PROs and further patient characteristics, we applied the Kruskal–Wallis test and Mann–Whitney U test as well to analyze correlations the Spearman’s correlation coefficient, respectively. We set the significance level to 5%.

## 3. Results

In total, a high response rate of 74% was obtained, with 82 patients (64 men, 78.0%; median age 71 years [IQR 65–78]) completing the survey. The detailed characteristics of the patient cohort are shown in Table 1. Most of the patients had been diagnosed according to clinico-pathologically defined IBM (*n* = 36, 43.9%), followed by clinically defined IBM (*n* = 28, 34.1%) and probable IBM (*n* = 18, 22%), respectively. The median for the age at symptom onset was 58 years and for the age at diagnosis 63.5 years. The median duration from misdiagnoses to a confirmed IBM diagnosis was 24 months (IQR 6–48). The majority of the patients were retired (*n* = 65, 80.2%) and married, living together with their partner (*n* = 67, 81.7%). The educational level and the percentage of private health insurance (32.5%) was in our study cohort slightly higher than the typical distributions in Germany [36,37]. Within the possible range (1–5) of the care levels applied in the German care system, 63.4% of the patients reported a care level of 2 or more, whereas 36.6% reported no care level at all. On the contrary, 84.5% of the patients reported a degree of disability (possible range 20–100), whereby most patients (*n* = 32, 41.6%) reported a degree between 60 and 90 compared to 21 patients (27.3%) with the highest possible degree of 100.

### 3.1. Physical Functioning, Comorbidities and Dysphagia

As Table 2 presents the collected PROs regarding clinical features, firstly, the physical function measured with the total sIFA score showed a median of 74 (IQR 42–88). For six out of the total eleven items a median of at least 8 was reported (‘stand from ordinary chair’, ‘get on and off toilet’, ‘walk outdoors’, ‘step up and down curbs’, ‘go up or down 5 steps’, ‘get up from the floor’). The two items regarding ‘swallow liquids/solids’ were reported with the lowest medians of 1 and 2, respectively. Secondly, patients reported having problems with a median of 2.5 comorbidities measured with the SCQ-D. In total, the median SCQ-D score was 5 (IQR 2–8). Thirdly, the most reported level of functional oral intake of food and liquids with the FOIS-G was ‘having oral diet with no restrictions’ (*n* = 51; 63%). Except for one patient being tube-dependent with consistent oral intake of food or liquids, all other patients reported a total oral diet. Fourthly, the second PROM to assess dysphagia showed a median total SSQ-G score of 192 (IQR 25.5–487). The median values for the subscales were 13 for the global scale, 179 for the physical scale and, lastly, 6 for the quality-of-life scale.

### 3.2. Descriptive Data of the Generic and Disease-Specific HRQoL and Mood

**Figure 1:**
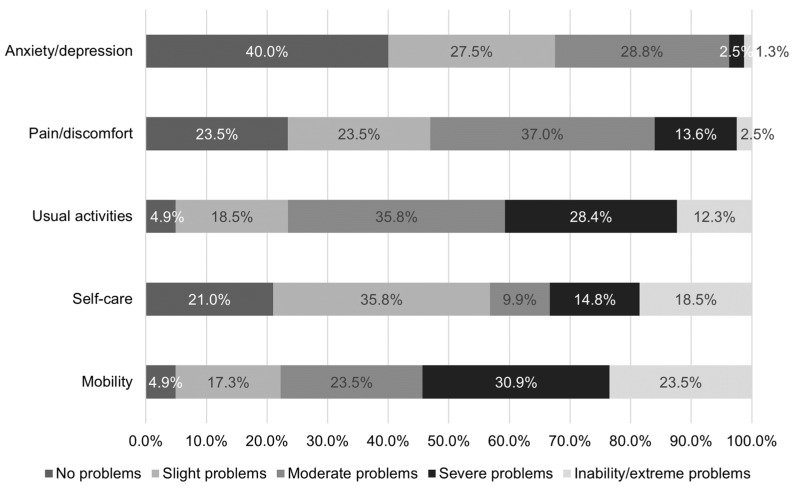
Proportion of responses by level of severity for EQ-5D-5L dimensions (*n* = 81; Anxiety/depression *n* = 80). Self-reported severity of problems for the EQ-5D-5L dimensions.

The patients rated their generic health today within the five dimensions of the EQ-5D. Two main points emerge from the data in Figure 1. Within the dimensions mobility and usual activities, IBM patients perceived most often severe to extreme problems (54.3%; 40.7%, respectively). Furthermore, only 3.8% experience severe to extreme problems regarding anxiety and depression and 16% regarding pain and discomfort, whereby the highest percentage for having no problems were also reported for anxiety and depression (40%), followed by pain/discomfort (23.5%) and self-care (21%). According to the German societal preferences, the mean EQ-5D index was 0.544 (SD 0.339). Moreover, the EQ-5D VAS as a simple instrument to quantitatively measure the self-reported generic overall health status on a scale from ‘best health you can imagine’ to ‘worst’ scored a mean of 48.7 (SD 21.6) in this IBM cohort.

The total score of the disease-specific assessed HRQoL in patients with NMDs, and thus IBM, was measured with the INQoL and showed a mean of 58.7 (SD 14.6). The symptoms with the most impact were weakness (mean 78.3, SD 16.1) and fatigue (mean 45, SD 30.6). The means of pain and dysphagia were similar (mean 36.1, SD 33.3; mean 33.7, SD 31.4, respectively). In addition, the subscale activities and independence impacted the life domains most (mean 69.1, SD 20.6; mean 61.9, SD 27.2, respectively). Furthermore, the means of the items regarding the treatment effects were quite low.

Turning to the self-reported mood scales, higher means were found for depression (mean 6.6, SD 3.7) than for anxiety (mean 5.9, SD 3.9). This is also reflected in the percentage of probably definite cases, as 19.5% of the patients (*n* = 16) scored more than 11 regarding depression (anxiety 13.4%, *n* = 11, respectively). While most of the IBM patients were suggested to have neither of these two psychiatric conditions (depression 62.2%; anxiety 64.6%, respectively), doubtful cases for depression were found in 18.3% and for anxiety in 22%. Details of the descriptive data regarding HRQoL and mood are given in Table 3.

### 3.3. Correlations between Disease-Specific HRQoL, Mood, Dysphagia and Physical Functioning

The correlations between the INQoL, HADS-D, SSQ-G and sIFA can be seen from Table 4. Strong positive correlations for the INQoL symptom scores were found for weakness and the sIFA, as well as for dysphagia and the SSQ-G. Weakness impact score was also moderately correlated with depression (HADS-D/D) and the SSQ-G (dysphagia). Other positive moderate correlations were found for fatigue impact score and anxiety and depression, as well as for dysphagia impact score and sIFA. Depression correlated with the INQoL life domains independence and emotions strongly, moderately with the remaining INQoL life domains (activities, social relationships and body image) as well as with the sIFA. In contrast, anxiety only correlated strongly with emotions and moderately with body image. Considering the physical functioning measured with the sIFA, the activities and independence subdomain showed strong correlations as well as moderate correlations with social relationships and body image. Dysphagia as measured with the SSQ-G was correlated moderately with the INQoL independence domain and strongly with the physical functioning (sIFA).

### 3.4. Differences Regarding Mood and Disease-Specific HRQoL

We further examined the PRO data of mood (HADS-D) and HRQoL (INQoL) for differences between sociodemographic and clinical features, as presented in Table 5. Firstly, we found no significant differences regarding anxiety. The highest means of anxiety showed the age group of 65–69 years (mean 6.9, SD 2.94), followed by female patients (mean 6.7, SD 3.495) and patients with a high educational level (mean 6.7, SD 3.951). Secondly, the only significant difference regarding depression was found for the reported degree of disability (*p* < 0.05). The pairwise comparison showed significant differences (*p* < 0.01) for the degrees of 20–50 (mean 4.5, SD 2.747) compared to 100 (mean 8.4, SD 4.166). Statistical significance was marginally missed for depression and disease duration (*p* = 0.054). Overall, the highest depression scales with a mean of 8.4 were reported for the subgroup of patients with a degree of 100 and with care level 5, suggesting that the mean of this patient group are doubtful depression cases. Thirdly, the considerable role of the degree of disability and care level becomes further apparent by the significant differences regarding the total INQoL score (*p* = 0.002 and *p* = 0.017, respectively). Patients with no degree of disability differ significantly with patients having a degree of disability of 100 (*p* < 0.01). Moreover, the subgroup of 20–50 shows significant differences compared to the group of 60–90 (*p* < 0.05) and 100 (*p* < 0.01). The significant differences regarding the care level were found within the pairwise comparisons for care level 4 and no care level (*p* < 0.01) and care level 2 (*p* < 0.5). The considerably highest INQoL mean value of 71.4 (SD 12.259) reported patients with care level 4, followed by patients aged 75–79 years (mean 66.3, SD 10.443) as well as for a degree of disability of 100 (mean 65.9, SD 15.144).

### 3.5. Utilization of Psychological Support Considering Mood

**Figure 2:**
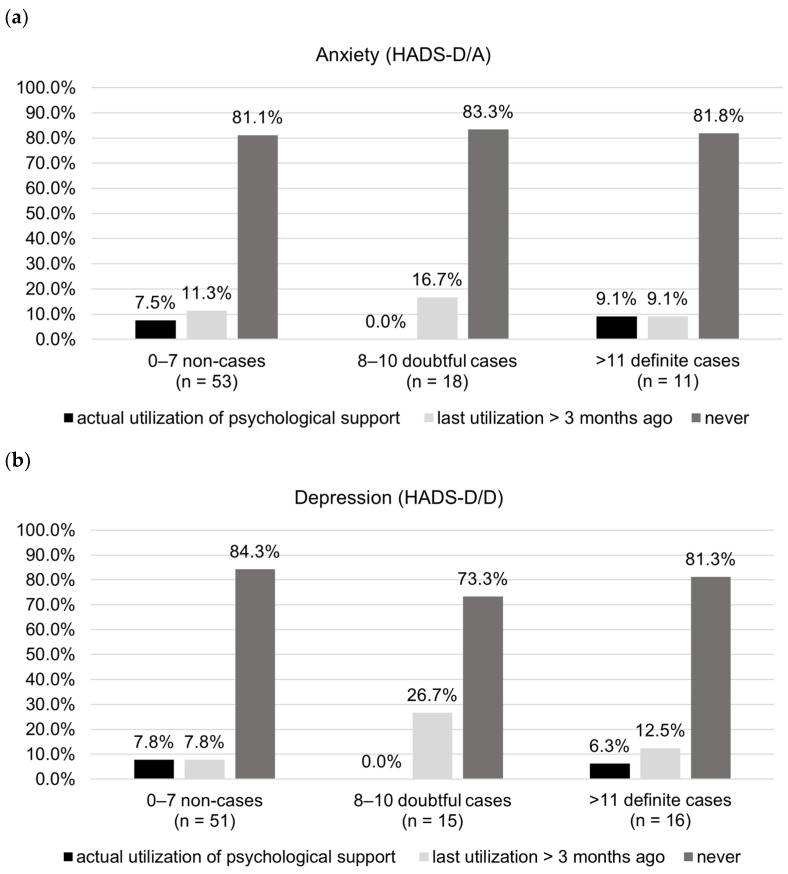
Resource utilization of psychological support (*n* = 82). (**a**) reported utilization of psychological support in relation to self-reported anxiety according to HADS, (**b**) reported utilization of psychological support in relation to self-reported depression according to HADS. Abbreviations: HADS-D/A = Hospital Anxiety and Depression Scale German version anxiety subscale; HADS-D/D = Hospital Anxiety and Depression Scale German version depression subscale.

The usage of psychological support is shown in Figure 2. Although more than half of the patients were no doubtful or definite anxiety or depression cases (Table 3), approximately 8% of this group actually utilized psychological support within the recall period of the last three months. However, within the subgroups of patients with doubtful or definite anxiety or depression, the majority never utilized psychological support (range: 73.3–83%). The data show that a past utilization of psychological support (>3 months ago) was minor-reported in doubtful or definitive cases, the most in doubtful depression cases (26.7%) and the least in definite anxiety cases (7.8%). Although there are some self-help group services for IBM patients existent in Germany, the patients reported to rather utilize professional psychological support than self-help group services.

## 4. Discussion

Our study provides for the first time the cross-sectional profiles of 82 German IBM patients regarding their generic and NMD-specific HRQoL as well as mental health using standardized and validated PROMs. In addition, we investigated the utilization of psychological support for identifying possible unmet needs. In summary, the physical dimension of HRQoL is suggested to be most affected in IBM patients, especially regarding weakness and fatigue. In addition, the patients’ mobility, independence and activities were mostly reported within the life domains using generic EQ-5D and the disease-specific INQoL, respectively. The screening for anxiety and depression was not noticeable for the majority of this IBM patient cohort. The highest possible degree of disability of 100 and the care level of 4 indicated significant differences regarding the total disease-specific HRQoL, the degree of disability of 100 regarding depression, respectively. Nevertheless, the mental health screening data further suggest that there might be an unmet need of psychological support in German IBM patients reporting doubtful or definite anxiety or depression.

Our findings are relevant for international comparisons using the sparse existing HRQoL and mental health data in IBM patients [41] in order to comprehensively identify or validate procedures as well as therapeutic options to optimize health-care delivery. Therefore, we extensively collected PRO data for subgroup analyses regarding the physical function and severity of dysphagia with the sIFA, SSQ-G and FOIS-G. As to our knowledge, no other study used such PROMs to better understand the HRQoL and mental health in IBM, thus our data could hardly be compared directly regarding physical function and dysphagia to other studies investigated in this research topic [17]. Audag et al. identified in their systematic review two studies evaluating dysphagia in IBM patients [42], whereby Olthoff et al. [43] detected in 80% dysphagia and impaired Swallowing-Related Quality of Life (SWAL-QoL) compared to reference values and Cox et al. [10] found patient-reported dysphagia symptoms in 65%. In contrast, almost the same number of patients reported in our study a total oral diet with no restrictions assessed with the FOIS-G. Taking into consideration that there is currently no published cut-off value for the German version of the SSQ [28] and also a comparable HRQoL study from Rose et al. [44] did not use the version 2.0 of the INQoL, in which the symptom dysphagia is included, we identified significantly strong correlations between the SSQ-G, the sIFA and INQoL dysphagia symptom impact. An ongoing prospective natural history study (NCT05046821) applies additionally to the sIFA also the SSQ for secondary outcomes measurements as well as not-further-specified HRQoL measures, which could increase the clinical knowledge of HRQoL data regarding dysphagia and physical function over disease progression. Remarkably, the sIFA as PROM for physical function in IBM patients only correlated strongly with INQoL weakness impact (ρ = 0.68) and with the life domains activities and independence (ρ = 0.65 and ρ = 0.79, respectively).

Furthermore, we identified a lower mean generic HRQoL (48.7, SD 21.6) as measured with the EQ-5D VAS in contrast to a study investigating differences regarding NT5c1A antibody (Mup44, CN1A) in IBM (mean seropositive 55; seronegative 65, respectively) [45], whereby the NT5c1A seronegative patients showed a comparable mean age and disease duration. Concerning the high simplicity of the EQ-5D VAS measurement and the respective median of 50 in our study population, this tendency to the middle could indicate the need of more disease-specific PROMs which better represent patient-relevant symptoms and life domains. Discussions are ongoing as to whether generic HRQoL measures as the EQ-5D are appropriate to capture disease-specific changes that really matter to the patients [46]. To our knowledge, no comprehensible data on the EQ-5D dimension have been reported in IBM patients. Annual cross-sectional PRO assessments in German IIM patients consider the five dimensions of the EQ-5D partly, only daily activities and anxiety/depression, and hence limit robust comparisons as there were previously only three IBM patients (2%) included in this IIM population [47]. Nevertheless, we found in IBM lower EQ-5D VAS mean values and more problems in the EQ-5D’s mobility, self-care and usual activities dimensions than in our previous cross-sectional study in patients with Charcot–Marie-Tooth (CMT) neuropathies (mean VAS 58.8, SD 19.8) [48].

Moreover, the obtained results valuably complement preliminary disease-specific HRQoL (INQoL) data of Rose et al., comprising 24 IBM patients [44]. Our identified means regarding the four symptoms covered in the first version of the INQoL were all lower, except for weakness (14.1% higher), with the highest difference regarding locking (19.6% lower). On the contrary, higher means were identified in our patient cohort for activities (11.1% higher), independence and social relationships (6.8% and 2.3% higher, respectively). Compared to the other investigated NMDs in this study [44] such as dermatomyositis/polymyositis (DM/PM), limb girdle muscular dystrophy (LGMD) facioscapulohumeral muscular dystrophy (FSHD) and other miscellaneous NMDs, the mean weakness impact score was only higher in FSHD (59.9, SD 28.1) than the mean measured in our IBM population. Interestingly, there are substantial differences between our means for locking (11.4, SD 23.9) and those reported from Rose et al. [44] in all investigated NMDs (min. mean 30.9 in IBM). Within our pretest, some patients mentioned that they have not understood the exact meaning of the INQoL symptom item ‘your locking’ (German translation ‘Ihre Muskel„starre”’), where myotonia should be assessed. This might be a reason for the low reported locking values in our sample and could indicate needed adjustments to the German INQoL translations. Besides comparisons on an item level, the mean total INQoL score in our IBM sample (mean 58.7, SD 14.6) was much higher than in patients with chronic inflammatory demyelinating polyradiculoneuropathy (CIDP; mean 43.6, SD 22.2) [49] and nearly twice as high than in other NMD patients [50]. Nevertheless, generic PROMs in order to assess HRQoL such as the Medical Outcomes Study 36-items Short Form (SF-36) [51] or the World Health Organization Quality of Life-BREF (WHOQOL-BREF) [52] are suggested to be more commonly used in IIM [41], and also IBM [17,53] though the INQoL has also been used in previous clinical trials (e.g., Rapamycin, NCT02481453) in IBM. Regarding the application of the INQoL version 2.0 as a PROM in the future clinical practice or research, the symptom item for dysphagia could generate valuable disease-specific knowledge about the impact of swallowing problems on the HRQoL in IBM patients, provided the strong correlations with the SSQ-G.

Furthermore, we described the disease-specific HRQoL data considering sociodemographic and clinical differences. An essential finding was that the data were heterogenous, whereby we identified two statistically significant differences for the total INQoL score: for the degrees of disability and the care levels applied in the German care system. Although not significant, a slightly worse disease-specific HRQoL could rather be associated with a medium education level, retirement, statutory health insurance, being single, using a wheelchair, being female and for the age group 75–79 years. Interestingly, the INQoL scores are lower in high-aged patients (>80 years), meaning better HRQoL than the age group 75–79 years. These findings support the inconsistencies regarding the role of age and disease severity on HRQoL among IIM and NMD patients [44,54,55].

In addition, the literature shows divergent reports regarding the psychological HRQoL in IBM [17]. Moderate correlations of anxiety and depression with the majority of the physical INQoL scores were found in NMD, comprising also IBM patients, as well as negative impairments of mental health, besides also observed no impairments and no significant differences of mental health in an IIM population [44,54,55]. By comparing our HADS-D data to an earlier study in myotonic dystrophy, both the anxiety and depression means were higher in IBM [56]. In sum, little is known about the disease-specific mental health in IBM patients until now, cross-sectional, as well as longitudinal. The low percentage of IBM patients in the respective studies comprising groups of NMD- or IIM patients limit therefore clear conclusions for IBM. However, our data allow to derive more detailed insights in the mental health of German IBM patients for the first time. The reported slight or even not-existing problems with anxiety/depression measured with the EQ-5D in 67.5% of the patients are in line with the HADS-D values, whereby 64.6% and 62.2% of the patients indicated no symptoms of anxiety and depression, respectively. Interestingly, if depression was measured with the SCQ-D by obviously asking the patients if they have the problem depression, fewer patients (*n* = 13, 15.9%) reported the comorbidity depression. It is noticeable that a further psychological evaluation in 35.4% of the patients classified with doubtful or definite anxiety (HADS-D/A) and 37.8% with doubtful or definite depression (HADS-D/D) should be undertaken in future clinical practice. Surprisingly, the degree of disability did only mildly correlate with depression but strongly with HRQoL. One possible explanation could be that the appropriate degree of disability has to be actively applied for by the patient in Germany. Patients with negative coping strategies might show a lack of action to apply for an appropriate degree of disability and may therefore be underrepresented in this classification. Thus, further research is needed to investigate the role of mental health and HRQoL in IBM.

By interpreting the self-reported utilization of psychological support, the relevance of need-oriented psychological support services could be highlighted by the fact that nearly four-fifths of this vulnerable subgroup have never utilized psychological support. Further research should therefore be undertaken for identifying valid screening tools for mental illnesses, e.g., depression, also for the application in IBM, such as the recommended Beck Depression Inventory II (BDI-II) [57] and Patient Health Questionnaire (PHQ-9) [58] for depression screening in rheumatoid arthritis [59]. The clinical practice should further foster preventive psychological support services, timely psychological diagnostics and initiate the according therapies to reduce patient burden and related societal costs besides the efforts to mitigate this progressive NMD. As IBM is a disease in the elderly, higher risks for depression such as widowhood, multiple metabolic problems or loneliness emphasize individual and constant follow-ups [60,61]. Thereby, it might be less relevant which medical speciality is performing such screenings or follow-ups rather than guaranteeing constant access to in-person or digital health-care services and other support services if the patient’s mobility and independence are severely limited.

In interpreting our findings critically, some limitations should be noted. Firstly, as we recruited IBM patients from a patient registry, these patients could be more dedicated and therefore imply a selection bias. Secondly, the patients in our sample showed a higher percentage with private health insurance and higher educational levels when compared to the general German population. More patients with a lower socioeconomic status could have resulted in higher psychological distress, greater risk for impaired HRQoL, greater social isolation and less financial margins to cope with the need of assistive devices and support services. Thirdly, restrictions within the COVID-19 pandemic could have resulted in reduced health-care provision and additional burden with regard to our recall period. Fourthly, we did not collect data on prescribed psychiatric medicine, only on self-reported utilized psychological support services. Thus, the conclusions regarding comprehensive guideline adherence in mental health pathologies are limited. In addition, brain disorders like frontotemporal dementia were not clinically assessed but nevertheless also not self-reported within the SCQ-D. Limitations regarding the cognitive abilities of this study sample are therefore rather unlikely.

## 5. Conclusions

In conclusion, for the first time our findings present detailed PRO data of the general and disease-specific HRQoL and mental health in German IBM patients. Physically, patients’ HRQoL is mostly limited due to weakness and fatigue, whereby socially, decreased independence and activities of daily living are most relevant. Although the majority of the IBM patients reported no symptoms for depression or anxiety, our data suggest possible unmet needs regarding psychological diagnostics and treatment. Furthermore, the degree of disability and the care level as used in the German health system imply to be relevant influencing factors for HRQoL and depression. Above all, PROMs could complement a holistic treatment evaluation or screening in clinical practice, in order to tailor health-care measures for more effective care resource allocations, thus improving outcomes and minimizing health inequalities.

## Figures and Tables

**Table 1:** Patient sociodemographic and health-related characteristics (*n* = 82).

Characteristics	*n* (%) or Median (IQR)
Male	64 (78.0)
Age	71 (65–78)
Age groups	
<65	19 (23.2)
65–69	13 (15.9)
70–74	20 (24.4)
75–79	15 (18.3)
>80	15 (18.3)
Age at symptom onset (*n* = 81)	58 (52–64)
Age at diagnosis	63.5 (58–69)
Duration from other diagnoses until IBM diagnosis in months (*n* = 35)	24 (6–48)
ENMC criteria ^a^	
Clinico-pathologically defined	36 (43.9)
Clinically defined	28 (34.1)
Probable	18 (22.0)
Marital status	
Single	1 (1.2)
Widowed	6 (7.3)
Divorced	6 (7.3)
Married, living apart	2 (2.4)
Married, living together	67 (81.7)
Employment status (*n* = 81)	
Retired	65 (80.2)
Non-working due to IBM	2 (2.5)
Employed	9 (11.1)
Self-employed	5 (6.2)
Educational level (*n* = 80) ^b^	
Low	1 (1.3)
Medium	46 (57.5)
High	33 (41.3)
Statutory health insurance (*n* = 80)	54 (67.5)
Care level ^c^	
No care level	30 (36.6)
Care level 1	0
Care level 2	18 (22.0)
Care level 3	20 (24.4)
Care level 4	9 (11)
Care level 5	5 (6,1)
Degree of disability ^d^ (*n* = 77)	
No degree of disability	12 (15.6)
20–50	12 (15.6)
60–90	32 (41.6)
100	21 (27.3)

Due to rounding, percentage might not add up to exactly 100%. Abbreviations: ENMC = European Neuromuscular Center; IQR = interquartile range. ^a^ Data gathered from IBM patient registry. ^b^ Educational level is reported according to the International Standard Classification of Education (ISCED) 2011 [38]. ^c^ In 2017, the definition of the need for care was revised in Germany. The extent of benefits from the German statutory care insurance are based on an individual score within six life domains of a person. A higher care level indicates a worse state of independence and capabilities [39]. ^d^ The degree of disability (20–100) is graduated in steps of 10. A higher degree indicates a higher level of physical, psychological or social disability [40].

**Table 2:** PROMs regarding physical functioning, comorbidities and dysphagia assessment.

	Median (IQR) or *n* (%)
**sIFA total (*n* = 81)**	74 (42–88)
Stand from ordinary chair	8 (4–10)
Get up from the floor	10 (8–10)
Get on and off toilet	8 (5–10)
Walk on a flat, firm surface	5 (3–8)
Walk outdoors	8 (5–10)
Go up or down 5 steps	9 (4–10)
Step up and down curbs	8 (4–10)
Swallow liquids	1 (0–3)
Swallow solids	2 (0–5)
Carry a 5-pound object	6 (3–10)
Grip and use small objects	6 (3–9)
**SCQ-D (*n* = 82)**	5 (2–8)
Problem	2.5 (1–4)
Treatment	1.5 (1–3)
Limited activities	0 (0–1)
**SSQ-G total (*n* = 81)**	192 (25.5–487)
Global (item 1)	13 (0–37.5)
Physical (item 2–16)	179 (25.5–429)
Quality of Life (item 17)	6 (0–30)
FOIS-G * (*n* = 81)	
Level 1: Nothing by mouth	0 (0)
Level 2: Tube-dependent with minimal attempts of food or liquid	0 (0)
Level 3: Tube-dependent with consistent oral intake of food or liquid	1 (1.2)
Level 4: Total oral diet of a single consistency	5 (6.2)
Level 5: Total oral diet with multiple consistencies but requiring special preparation or compensations	5 (6.2)
Level 6: Total oral diet with multiple consistencies without special preparation, but with specific food limitations	18 (22.2)
Level 7: Total oral diet with no restrictions	51 (63.0)
n/a	1 (1.2)

Due to rounding, percentage might not add up to exactly 100%. Abbreviations: FOIS-G = Functional Oral Intake Scale German version; IQR = interquartile range; sIFA = sIBM Physical Functioning Assessment; SCQ-D = German Self-Administered Comorbidity Questionnaire; SSQ-G = Sydney Swallow Questionnaire German version. * We applied the German Version (FOIS-G) in our survey; English items of FOIS are displayed in this figure to ensure readability.

**Table 3:** INQoL Profile Scores (*n* = 79), HADS-D Scores (*n* = 82) and EQ-5D-5L (Index *n* = 80; VAS *n* = 82).

PROM	Dimensions	Subscales	Median (IQR) or *n* (%)	Mean (SD)
EQ-5D- 5L	EQ-5D Index		0.635 (0.372–0.801)	0.544 (0.339)
	EQ VAS		50 (35–62.8)	48.7 (21.6)
INQoL	Total score	Quality of life	58 (51–68)	58.7 (14.6)
	Symptoms	Weakness	79 (68.4–84.2)	78.3 (16.1)
		Pain	47.4 (0–68.4)	36.1 (33.3)
		Locking	0 (0–0)	11.3 (23.9)
		Fatigue	52.6 (15.8–68.4)	45 (30.6)
		Ptosis	0 (0–0)	2 (8.3)
		Diplopia	0 (0–0)	2.9 (11.4)
		Dysphagia	36.8 (0–63.2)	33.7 (31.4)
	Life domains	Activities	75 (56–86)	69.1 (20.6)
		Independence	67 (39–86)	61.9 (27.2)
		Social relationships	31 (19–49)	35.1 (20.9)
		Emotions	33 (22–58)	38.8 (22.6)
		Body image	53 (31–64)	50.1 (22.3)
	Treatment effects	Perceived treatment effects	17 (0–50)	24.5 (28.7)
		Expected treatment effects	8 (0–50)	20.3 (27.9)
HADS-D	Anxiety	HADS-D/A	5.5 (3–9)	5.9 (3.9)
		0–7 non-cases	53 (64.6)	
		8–10 doubtful cases	18 (22)	
		>11 definite cases	11 (13.4)	
	Depression	HADS-D/D	6 (3.8–10)	6.6 (3.7)
		0–7 non-cases	51 (62.2)	
		8–10 doubtful cases	15 (18.3)	
		>11 definite cases	16 (19.5)	

Due to rounding, percentage might not add up to exactly 100%. Abbreviations: EQ-5D = European Quality of Life 5 Dimensions; HADS-D = Hospital Anxiety and Depression Scale German version; HADS-D/A = subscale anxiety; HADS-D/D = subscale depression; IQR = interquartile range; INQoL = The Individualized Neuromuscular Quality of Life; SD = standard deviation; VAS = visual analogue scale.

**Table 4:** Correlations between the PROMs regarding HRQoL, mental health, dysphagia and physical function (ρ-values).

		HADS-D		SSQ-G	sIFA
		Anxiety	Depression		
INQoL Symptoms	Weakness	0.12	0.43 **	0.32 **	0.68 **
	Pain	0.29 **	0.4 **	0.15	0.4 **
	Locking	0.23 *	0.2	0.1	0.17
	Fatigue	0.36 **	0.36 **	0.23 *	0.29 **
	Dysphagia	0.26 *	0.24 *	0.88 **	0.47 **
INQoL Life Domains	Activities	0.15	0.47 **	0.26 *	0.65 **
	Independence	0.14	0.5 **	0.36 **	0.79 **
	Social relationships	0.16	0.35**	0.19	0.35 **
	Emotions	0.66 **	0.7 **	0.16	0.27 *
	Body image	0.3 **	0.38 **	0.29 **	0.43 **
HADS-D	Anxiety		0.71**	0.29 **	0.14
	Depression	0.71 **		0.27 *	0.43 **
SSQ-G		0.29 **	0.27 *		0.52 **
sIFA		0.14	0.43 **	0.52 **	

Abbreviations: EQ-5D = European Quality of Life 5 Dimensions; HADS-D = Hospital Anxiety and Depression Scale German version; INQoL = The Individualized Neuromuscular Quality of Life; sIFA = sIBM Physical Functioning Assessment; SSQ-G = Sydney Swallow Questionnaire German version; VAS = visual analogue scale. * *p* < 0.05, 2-tailed. ** *p* < 0.01, 2-tailed.

**Table 5:** Clinical and sociodemographic differences regarding mental health and disease-specific HRQoL.

	Anxiety				Depression			INQoL Total Score		
	*n*	Mean	SD	*p*	Mean	SD	*p* (Corr. *p* ^a^)	Mean	SD	*p* (Corr. *p* ^a^)
Age groups				0.494			0.506			0.149
<65	19	5.0	3.801		5.5	3.272		54.0	14.937	
65–69	13	6.9	2.940		6.1	3.148		57.9	11.796	
70–74	20	6.3	3.210		6.6	3.575		56.9	15.712	
75–79	15	6.5	4.998		7.9	4.324		66.3	10.443	
>80	15	5.1	4.580		7.2	4.178		59.5	16.479	
Age				0.966			0.074			0.131
Sex				0.216			0.942			0.15
Male	64	5.7	4.029		6.7	3.851		57.36	15.0	
Female	18	6.7	3.495		6.4	3.258		62.8	12.081	
ENMC criteria				0.454			0.487			0.233
Clinico-pathologically defined	36	5.3	3.567		6.1	3.798		56.2	12.906	
Clinically defined	28	6.2	3.794		7.0	3.305		62.9	13.538	
Probable	18	6.6	4.767		7.1	4.185		57.0	17.965	
Degree of disability				0.945			0.045 *			0.002 **
No degree of disability (100 ^b^)	12	6.1	4.852		5.9	3.753		50.6	14.89	0.004 **^a^ (0.024 *)
20–50 (100 ^b^)	12	5.4	3.825		4.5	2.747	0.006 **^a^ (0.036 *)	48.6	14.394	<0.001 **^a^ (0.005 **)
20–50 (60–90 ^b^)										0.041 *^a^ (0.244)
60–90	32	5.8	3.610		6.6	3.491		59.9	11.822	
100	21	6.5	4.343		8.4	4.166		65.9	15.144	
Disease duration (since first symptoms)				0.171			0.054			0.175
Wheelchair use				0.777			0.173			0.363
Yes	31	5.8	3.842		7.4	3.844		59.8	15.829	
No	51	5.9	4.007		6.1	3.583		57.8	13.764	
Health insurance				0.585			0.849			0.951
Private	26	5.5	4.178		6.5	4.062		57.9	17.33	
Statutory	54	6.0	3.873		6.6	3.61		58.5	13.176	
Care level				0.859			0.071			0.017 *
No care level (Care level 4 ^b^)	30	5.9	3.933		5.0	2.883		53.1	12.448	<0.001 **^a^ (0.007 **)
Care level 1	0									
Care level 2 (Care level 4 ^b^)	18	5.5	3.634		7.3	3.430		58.7	11.961	0.042 *^a^ (0.418)
Care level 3	20	6.5	4.286		7.4	3.775		61.4	16.671	
Care level 4	9	6.1	4.076		8.0	4.137		71.4	12.259	
Care level 5	5	4.4	4.159		8.4	5.771		56.0	16.492	
Marital status				0.848			0.858			0.42
Single, widowed, divorced	13	6.0	3.937		6.4	3.404		61.9	17.762	
Married	69	5.9	3.948		6.7	3.788		57.9	13.887	
Employment status				0.158			0.222			0.166
Retired	65	6.3	3.906		6.9	3.706		60.1	14.449	
Non-working due to IBM	2	1.0	1.414		2.5	2.121		50.5	2.121	
Employed	9	4.6	3.844		5.7	3.0		56.3	8.426	
Self-employed	5	6.2	3.899		5.0	3.391		44.6	2.303	
Educational level				0.328			0.135			0.064
Medium	46	5.5	3.846		7.0	3.509		61.0	13.067	
High	33	6.7	3.951		5.9	3.726		54.3	13.946	

Abbreviations: ENMC = European Neuromuscular Center; HADS-D = Hospital Anxiety and Depression Scale German version; INQoL = The Individualized Neuromuscular Quality of Life, overall composite score; *p* = *p*-value; SD = standard deviation; ^a^ *p*-values after Bonferroni correction are additionally reported in brackets for pairwise comparison of variables showing significant differences. ^b^ Variables with significant pairwise comparisons. * *p* < 0.05. ** *p* < 0.01.

## Data Availability

The data presented in this study are available on request from the corresponding author.
